# Evaluating ChatGPT-4 Plus in Ophthalmology: Effect of Image Recognition and Domain-Specific Pretraining on Diagnostic Performance

**DOI:** 10.3390/diagnostics15141820

**Published:** 2025-07-19

**Authors:** Kevin Y. Wu, Shu Yu Qian, Michael Marchand

**Affiliations:** 1 Department of Surgery, Division of Ophthalmology, University of Sherbrooke, Sherbrooke, QC J1G 2E8, Canada; 2 Faculty of Medicine, University of Sherbrooke, Sherbrooke, QC J1G 2E8, Canada

**Keywords:** ophthalmology, ChatGPT, artificial intelligence, accuracy, pretraining

## Abstract

**Background/Objectives**: In recent years, the rapid advancements in artificial intelligence models, such as ChatGPT (version of 29 April 2024), have prompted interest from numerous domains of medicine, such as ophthalmology. As such, research is necessary to further assess its potential while simultaneously evaluating its shortcomings. Our study thus evaluates ChatGPT-4’s performance on the American Academy of Ophthalmology’s (AAO) Basic and Clinical Science Course (BCSC) Self-Assessment Program, focusing on its image recognition capabilities and its enhancement with domain-specific pretraining. **Methods**: The chatbot was tested on 1300 BCSC Self-Assessment Program questions, including text and image-based questions. Domain-specific pretraining was tested for performance improvements. The primary outcome was the model’s accuracy when presented with text and image-based multiple choice questions. Logistic regression and post hoc analyzes examined performance variations by question difficulty, image presence, and subspecialties. **Results**: The chatbot achieved an average accuracy of 78% compared with the average test-taker score of 74%. The repeatability kappa was 0.85 (95% CI: 0.82–0.87). Following domain-specific pretraining, the model’s overall accuracy increased to 85%. The accuracy of the model’s responses first depends on question difficulty (LR = 366), followed by image presence (LR = 108) and exam section (LR = 79). **Conclusions**: The chatbot appeared to be similar or superior to human trainee test takers in ophthalmology, even with image recognition questions. Domain-specific training appeared to have improved accuracy. While these results do not necessarily imply that the chatbot has the comprehensive skill level of a human ophthalmologist, the results suggest there may be educational value to these tools if additional investigations provide similar results.

## 1. Introduction

Artificial intelligence (AI) models, such as ChatGPT, have recently shown tremendous potential in an assortment of medical fields, including ophthalmology. These models comprise a rapidly evolving field of technologies capable of accomplishing complex human-level tasks and extracting abstract patterns from real-world datasets [1]. In recent years, the integration of AI technologies in various fields, including healthcare, has revolutionized conventional approaches to problem-solving and decision-making. This includes a plethora of applications, ranging from clinical decision support systems to automating writing processes in medical education and research [2,3]. In the field of ophthalmology, AI has been implemented to aid with diagnostic decision making, such as through ocular imaging recognition and analysis, with some models showing a performance similar to that of experienced ophthalmologists [4,5]. Another application of note is natural language processing, where AI tools are able to parse and generate textual information to generate discharge summaries and operative notes [6,7].

ChatGPT, a natural language processing chatbot developed by OpenAI, is one such tool that has witnessed remarkable advancements since its inception. Powered by a large-language model (LLM), ChatGPT employs deep learning techniques trained on vast amounts of data to generate dialogue and answer questions in a human-like conversation manner [8]. The generative pre-trained transformer (GPT) large language model architecture was first introduced in 2018, and subsequent interactions have led to the development of ChatGPT-4 Plus in 2024, which now has the ability to analyze image inputs and allow its users to customize and train their own GPTs on domain-specific knowledge [9]. This increasing popular chatbot reached 800 million active weekly users in April 2025 and processes more than one billion queries on a daily basis [10]. Specific applications of ChatGPT in ophthalmology include generating explanations for patient education, automating medical administrative and research tasks, and creating or answering practice questions for medical learners [11,12,13]. However, potential limitations include a lack of transparency and explanation in the way responses are generated, data security, and concerns about ChatGPT’s potential to provide harmful and incorrect information in areas like medicine [11,14]. With human supervision and future iterations, ChatGPT may become a valuable tool for clinicians. Nevertheless, further research into evaluating its clinical accuracy is needed.

Evaluation of ChatGPT’s ophthalmology accuracy in the current literature has shown promising results, but are limited by a lack of research in ChatGPT’s new image processing capacity. In terms of diagnostic capability, Madadi et al., as well as Delsoz et al., demonstrated that ChatGPT-4 had diagnostic accuracies greater than 80% when provided with ophthalmology case report information [15,16]. Comparatively, this was a significant improvement in performance relative to ChatGPT-3.5, which showed accuracies in the range of 50–60% [15,16,17]. ChatGPT was able to generate responses and complete the task 4–5 times faster than residents and attendings, demonstrating its potential utility in expediting clinical decision-making processes. Multiple papers have also evaluated ChatGPT’s accuracy at answering ophthalmology and medical board exam questions such as the United States Medical Licensing Examination (USMLE). Haddad et al. extracted ophthalmology-related questions from the USMLE and other sources, and found that ChatGPT-3.5 and ChatGPT-4 achieved 55% and 70% correct answer rates respectively [18]. Sakai et al. employed a similar methodology for evaluating questions from Japanese board exams; however, they found that the performance of ChatGPT-4 decreased to 45.8%, which indicates that there is some variability in performance in the literature [19]. Using the Basic and Clinical Science Course (BCSC) Self-Assessment Program and OphthoQuestions question bank, Antaki et al. illustrated that, prior to the release of ChatGPT-4, ChatGPT-3.5 achieved 55.8% and 42.7% accuracy, respectively, on questions from these tests that did not include images [20]. Mihalache et al. showed a similar result with a correct answer rate of 46% on the OphthoQuestions set [21]. Overall, ChatGPT has shown moderate accuracy, ranging from 40–70%, when answering ophthalmology-related questions. All of these studies also stratified their results, with ChatGPT mostly performing better on general medicine and easier questions, which had a higher correct answer rate amongst human participants. To our knowledge, previous studies have mainly evaluated ChatGPT-4’s performance on text-based questions.

As a result, the primary objective of this study is to assess ChatGPT-4 Plus’s accuracy at responding to the BCSC ophthalmology questions, inclusive of clinical imagery, in comparison with the average test-taker’s performance. Secondary objectives include assessing the repeatability of ChatGPT-4 Plus’s performance, differentiating performance based on disciplines, image presence and difficulty, and evaluating the improvement of ChatGPT-4 Plus with domain-specific pretraining. The main novelties of our study include the examination of image-based questions and comparisons between pretrained and non-pretrained versions of the chatbot. Another contribution of our study to the literature is the incorporation of a larger number of questions since previous reports often utilized much smaller sample sizes [19,22,23]. Evaluation of this performance will help elucidate the potential implications of implementing ChatGPT-4 Plus for clinical diagnostics and medical education in ophthalmology.

## 2. Materials and Methods

Our study aimed to evaluate the capabilities of ChatGPT-4 Plus (Open AI, San Francisco, CA, USA), focusing on its performance in answering questions sourced from the BCSC Self-Assessment Program (AAO, San Francisco, CA, USA). The primary objective was to assess the model’s accuracy, incorporating both text and image-based questions. Secondary objectives included the following: evaluating its repeatability; differentiating performance based on disciplines, image presence, and difficulty; and determining the effect of domain-specific pretraining on its accuracy.

Our study focused on ChatGPT-4 Plus (version of 29 April 2024), an advanced version in the ChatGPT series. This iteration is notable for its ability to process and interpret visual information in addition to text. Furthermore, it introduces the capability for domain-specific pretraining by allowing the creation of new custom GPT models. These models are trained using BCSC textbooks, which involved creating a new ChatGPT instance and uploading textbooks through the “knowledge” configuration in the settings. Individual GPTs were created for each testing category and only contained the materials corresponding to its subspecialty.

Data collection involved extracting a dataset of 1300 questions from the BCSC Self-Assessment Program, which included both text-based and image-based questions. The BCSC question bank contains 13 testing categories, and 100 questions were randomly selected from each section via the website’s testing set generation function. This process was carried out by three operators who carefully copied and pasted each question (with the corresponding images, if any) into ChatGPT-4 Plus, ensuring that the original formatting of the test questions was preserved. Each question was fed into ChatGPT4 one at a time and in a manner that initiated a new session for each query, eliminating any potential memory retention bias. The lead-in prompt used was: “Please provide the right answer.” To evaluate repeatability, each question was administered three times, and responses from ChatGPT-4 Plus were then compared with the official answers to gauge accuracy. Due to the AI’s privacy policies, it sometimes refused to analyse photos containing facial features. In those situations, that question was removed and a new question was randomly selected to take its place. This process involved operators filling out a data collection sheet (an Excel file) that recorded the correct answer (as provided by the question bank) against the responses from ChatGPT. This included four sets of answers: three from repeated sessions with ChatGPT-4 Plus and one from the session with ChatGPT-4 Plus with domain-specific pretraining. Additionally, this sheet captured other relevant variables, such as subspecialties, difficulty level, and the presence or absence of images. To further refine our analysis, a subgroup study was undertaken to assess how these variables—particularly the inclusion of images, subspecialty content, and question difficulty—impacted performance. Separate spreadsheets were created for each variable. The thresholds used to classify question difficulty—≥70% success rate as easy, between ≥30% and <70% as moderate, and <30% as difficult—were adopted based on previously published studies. This classification reflects established conventions in the literature for categorizing question difficulty according to empirical success rates [20]. These success rates were directly taken from the values reported on the BSCS website.

To evaluate the accuracy of ChatGPT, we compared its responses to the correct answers from the question banks. This analysis was performed on the ChatGPT-4 model, where accuracy assessments were conducted over three separate runs. From these, we calculated the mean accuracy rates. To measure the consistency of these runs across different sections of the examination, we used unweighted Cohen’s kappa statistic for multiple raters (κ measures).

Our investigation also included a logistic regression analysis, incorporating all input variables simultaneously to assess the impact of the examination section, the inclusion of images, and the question difficulty level on the accuracy of ChatGPT’s responses. Following this, multiple comparisons were conducted using False Discovery Rate adjustment. This was performed to identify any significant discrepancies in accuracy across the examination sections, while adjusting for variations in question difficulty and the presence of images. This method allowed us to isolate and examine the influence of the examination section on accuracy, determining any substantial differences among the topics tested. Results are presented as means and 95% confidence interval. Further details on the formula used in our analyses are detailed in Appendix A.

A minimum sample size calculation was conducted to achieve a confidence level of 95%. Based on prior studies evaluating AI accuracy in medical question banks, we anticipated a baseline accuracy of approximately 75% with a 5% margin of error. Using a standard formula for proportions, n = (Z^2^ × *p* × (1 − *p*))/*E*^2^, where *Z* corresponds to the 1.96 critical value for a 95% confidence level, *p* is the expected proportion (0.75), and *E* is the margin of error (0.05), the minimum required sample size was calculated to be 288 questions. Given that our study included 1300 questions—over four times the minimum—distributed equally across all 13 examination sections, the sample size provides ample power to detect statistically and clinically meaningful differences in performance between models.

For analyses concerning the ChatGPT Plus model with domain-specific pretraining, only a single run was executed. Statistical analyzes were conducted with R v.4.0.2 (R Core Team, Vienna, Austria). Given the numerous evaluations undertaken, some of which were post hoc to the initial evaluation, all *p*-values should be considered nominal for hypothesis generation, and not for statistical significance. Ethical approval was not required as the study does not involve human or animal subjects, as confirmed by the ethics board of the University of Sherbrooke’s Research Center, Canada.

## 3. Results

### 3.1. Baseline Characteristics of the Testing Sets

The baseline characteristics of the testing sets are classified by difficulty level and by the presence of images across various ophthalmology sections. Table 1 details the distribution of 1300 questions as follows:

- Questions are categorized by difficulty: easy, moderate, and difficult.
- The presence of images in questions is noted as either with or without images.

The subspecialties covered included general medicine, fundamentals, clinical optics, ophthalmic pathology, neuro-ophthalmology, pediatrics and strabismus, oculoplastics, cornea, uveitis, glaucoma, lens and cataract, retina and vitreous, and refractive surgery, each with 100 questions. Across the 13 BCSC sections, there was an uneven distribution of both question difficulty and presence of images. For example, some sections, such as ophthalmic pathology and clinical optics, featured a higher number of difficult questions, while sections such as oculoplastics and cornea recorded the lowest quantity of difficult questions. As for presence of images, cornea and retina and vitreous were the sections with the greatest number of questions with images, while general medicine and fundamentals had the lowest number.

### 3.2. Repeatability of ChatGPT Performance

A κ value of 0.61–0.80 indicates substantial agreement, while values above 0.80 suggest almost perfect agreement, demonstrating strong, reliable consistency beyond chance [24]. The overall Cohen’s kappa coefficient is 0.85 (95% confidence interval [CI], 0.82–0.87), as shown in Table 2, indicating its nearly perfect consistency to identical questions. The κ values for individual sections ranged from 0.79 to 0.95.

### 3.3. Comparative Performance Analysis of ChatGPT Models and Average Test-Takers Across Examination Sections

**Figure 1:**
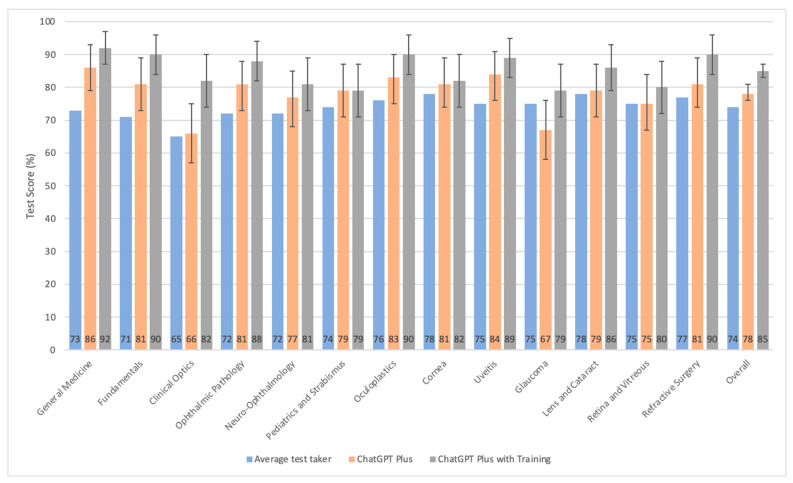
Bar graph comparing the test scores of the human test-takers, the non-pretrained, and pretrained model across examination sections. Error bars represent 95% confidence intervals.

As shown in Figure 1, ChatGPT Plus’s ability to match or to outperform the average test taker extends across all examination sections except for glaucoma. More precisely, the most marked areas in which the ChatGPT Plus model showed statistically superior scores compared to average test takers were general medicine, fundamentals, uveitis, and ophthalmic pathology, and to a lesser degree, neuro-ophthalmology, pediatrics and strabismus, oculofacial plastics, and refractive surgery sections. For the clinical optics, in the lens and cataract as well as the retina and vitreous sections, the model achieved scores similar to the average test taker. Overall, across all sections, ChatGPT Plus model outperformed the average test taker by 4% (95% I 2–7%).

When comparing the performance of the ChatGPT Plus model to that of the domain-specific pretrained ChatGPT Plus model, there is a clear trend of improvement with the introduction of domain-specific training. Notably, the pretrained model demonstrated a significant enhancement in accuracy in most of the sections, particularly in clinical optics, oculofacial plastics, glaucoma, and refractive surgery. When all sections are combined, the domain-specific pretrained ChatGPT Plus model outperformed the non-trained ChatGPT Plus model by 7% (95% CI 5–9%). The domain-specific pretrained ChatGPT Plus model also outperformed in all sections of the test, with exception of pediatrics and strabismus, where the performance was identical.

Overall, across all sections, the domain-specific pretrained ChatGPT Plus model outperformed the average test taker by 11% (95% CI 9–13%). The most substantial differences in performance are observed in the general medicine, fundamentals, clinical optics, ophthalmic pathology, oculofacial plastics, and uveitis sections, with the AI models demonstrating a clear advantage.

### 3.4. Performance Metrics Across Different Difficulty Levels

**Figure 2:**
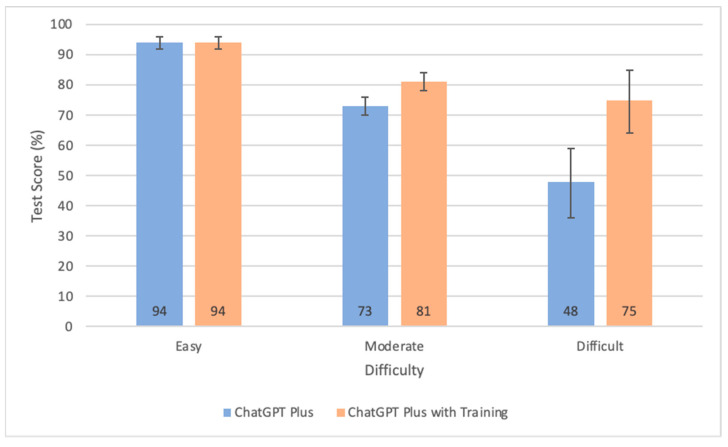
Bar graph comparing the test scores of the non-pretrained and pretrained models categorized by difficulty levels. Error bars represent 95% confidence intervals.

In the comparison between ChatGPT Plus and ChatGPT Plus with pretraining, both models demonstrated the same accuracy rate of 94% (95% CI 92–96%) for questions categorized as easy, as seen in Figure 2. However, distinctions became evident when questions got harder, with the pretrained model exhibiting higher test scores. For moderate difficulty questions, ChatGPT Plus with pretraining achieved a test result of 81% (95% CI 78–84%) compared to the non-pretrained model’s result of 73% (95% CI 70–76%). These differences became even more pronounced for difficult questions, with the pretrained model attaining a test score of 75% (95% CI 65–86%), as opposed to its counterpart’s score of 48% (95% CI 37–60%).

### 3.5. Comparative Analysis of Test Scores Based on the Presence of Images

In comparing ChatGPT Plus with and without pretraining, the pretrained version significantly outperformed in text-only questions, scoring 89% (95% CI: 87–91%) compared to 81% (95% CI: 79–83%) for the non-pretrained. Conversely, in questions with images, the pretrained version paradoxically underperformed at 55% (95% CI: 47–63%) versus 58% (95% CI: 50–66%) for the non-pretrained, though this difference wasn’t statistically significant.

## 4. Discussion

### 4.1. Comparative Review of ChatGPT’s Performance in Ophthalmology Assessments

In our study, we evaluated the performance of our ChatGPT Plus 4.0 model on the BCSC self-assessment questions bank. The model achieved an average score of 78%, and an enhanced domain-specific pretrained model scored 85%. These results are significantly higher than the average human test taker, who scored 74% overall.

Recent investigations have focused on the efficacy of ChatGPT in responding to queries related to ophthalmology, leveraging the question bank from BCSC self-assessment and OphthoQuestions. A notable study by Antaki et al. highlighted that ChatGPT’s weakest performance areas were optics and glaucoma, achieving a 72.9% accuracy rate [25]. This observation aligns with our study, which also identified weaknesses in those same sections. In a similar vein, Cai et al. found that GPT-4’s accuracy was 71.6% when tested with BCSC questions, closely aligning with Antaki’s findings [26]. Additionally, Mihalache and colleagues performed a similar study, utilizing a limited dataset from OphthoQuestions, and reported a higher accuracy rate of 84% [27]. This increase might be misleading due to the use of publicly available questions, justifying the improved performance in this particular study. Teebagy et al. also assessed GPT-4 using the BCSC question set and reported an 81% accuracy rate [28].

The variation in GPT-4’s reported accuracy rates in those previous studies can be attributed to several factors, including differences in the datasets used (varying in difficulty and cognitive demand), the comparison between BCSC and OphthoQuestions (with BCSC questions typically yielding higher scores) [20], and the inherent fluctuation in the model’s performance over time [29].

### 4.2. Performance Across Subspecialties, Question Difficulties and Image Presence

We found that ChatGPT-4’s answer accuracy depends first on question difficulty (LR = 366), followed by image presence (LR = 108), and then exam section (LR = 79). ChatGPT received higher scores on questions that are classified as easy, without images, and from particular sections (e.g., general medicine, fundamental).

This study distinguishes itself as the first to incorporate questions accompanied by images, unlike prior research that solely included text-based questions [20,25,26,27,28]. In our investigation, the integrated images encompass clinical visuals of the ocular surface and anterior segment, fundoscopy, radiographs (e.g., OCT, CT scans, and MRI), and visual fields, as well as clinical images of various body parts (such as the periocular structure, face, hands, feet, and skin).

**Figure 3:**
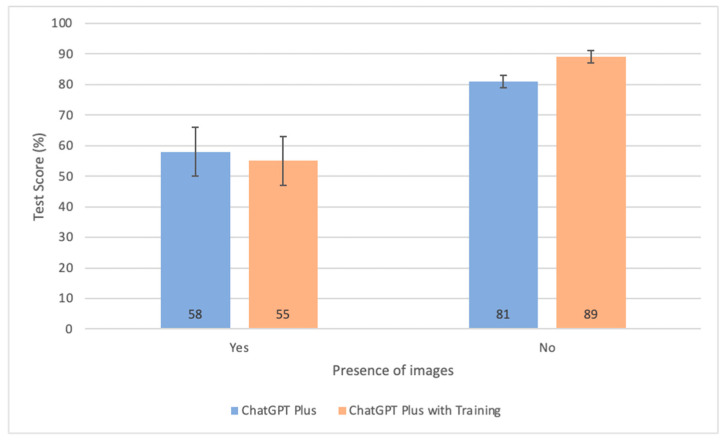
Bar graph comparing the test scores of the non-pretrained and pretrained models categorized by presence or absence of images. Error bars represent 95% confidence intervals.

Regarding the inclusion of images, both models demonstrated significantly lower performance scores when questions were accompanied by images, as shown in Figure 3. The detrimental impact of image presence on performance can be attributed to multiple factors. Firstly, ChatGPT has limited visual interpretation capabilities by design due to its innate text-centric architecture despite its recent steps towards multimodal processing abilities [30]. Therefore, concurrently analyzing text and images may be challenging for an AI model unequipped to handle both modalities. Furthermore, there may be a scarcity of pretraining data consisting of clinical images within the public domain and open-source databases used to train ChatGPT. In fact, ChatGPT was primarily trained on large quantities of text data and doesn’t directly learn from visual content even though it may have some ability to handle textual descriptions of images [31]. As such, the model might have more difficulty correlating visual patterns with medical concepts, thus making it less reliable when a prompt required direct visual understanding. Additionally, questions involving images may be intrinsically more complex because of the large quantity of information contained inherently within the image on top of the already present information from the text. Another aspect to consider is the fact that the interpretation of images by ChatGPT necessitates a substantial number of tokens, attributed to the increased complexity and the significant allocation of tokens required for image analysis. This can inadvertently result in a diminished token reservoir for the critical tasks of interpreting, reasoning, and generating responses based on its embedded knowledge [32]. The performance of the pretrained model paradoxically decreases in questions with images, suggesting that the extensive tokens required for database navigation might compromise the model’s capacity for effective image analysis, or vice-versa.

Even though images significantly impact ChatGPT’s accuracy, this challenge signals the beginning of a new era in generative AI, introducing multimodal capabilities. This advancement is especially pertinent in ophthalmology, a field heavily reliant on imaging for diagnosis and monitoring. The future lies in integrating LLMs with systems capable of processing multimodal inputs, thereby enhancing their practical utility in medical applications [33]. To improve image recognition capabilities, the model should utilize large datasets of images, particularly in combination with domain-specific fine-tuning to assist the AI in its understanding of the images’ context [34]. Moreover, hybrid chatbots that combine LLMs with AI models specialized for image analysis, such as convolutional neural networks, may demonstrate enhanced performance [35]. Lastly, allowing for feedback loops between the AI and its users during its training processes would increase the model’s adaptability while constantly refining its accuracy [36].

Among the 13 sections, the AI achieved various accuracy rates, ranging from 66% to 86% for the non-pretrained model and from 79% to 92% for its pretrained counterpart. This study reaffirms findings from previous research indicating that AI performance is better in general areas, such as general medicine and fundamentals, compared to more specialized fields, like glaucoma, retina, neuro-ophthalmology, and clinical optics, even after controlling for other factors, such as varying difficulty levels and inclusion of images [20,25,26,27,28]. Antaki et al. proposed that this disparity in performance could stem from the broader comprehension and simpler nature of general topics, unlike specialized subjects that demand intricate knowledge possibly beyond the grasp of general ophthalmology experts [20]. Variations in AI performance, exemplified by ChatGPT, potentially mirror the range of human expertise levels, which may in turn relate to the accessibility and caliber of online information. The training of ChatGPT and comparable models on extensive internet datasets means that their expertise in specific fields aligns with the relative online abundance of those topics. On the one hand, general areas, compared to specialized ones, endowed with a wealth of online resources, enable AI to thrive by leveraging a vast pool of training data. On the other hand, specialized fields might witness diminished AI efficacy due to the limited availability of comprehensive, specialized training materials [20,25]. Moreover, sections like general medicine typically rely on single-step decision-making, which contrasts sharply with fields such as clinical optics and glaucoma. The latter, often requiring simultaneous recall and complex decision-making, pose a more significant challenge, leading to lower performance outcomes—an empirical observation, albeit not quantitatively assessed in our study.

Although our study did not categorize questions by cognitive level, we intuitively observed that questions requiring lower cognitive effort, akin to recall tasks, resulted in higher performance compared to those necessitating complex clinical decision-making. This pattern suggests that ChatGPT-4 excels at tasks based on memorization, indicating potential limitations in its capacity for advanced reasoning. Antaki et al. have empirically demonstrated the impact of cognitive level on ChatGPT’s performance [25].

In terms of the effect of difficulty level on performance, the analysis of performance metrics reveals an inverse relationship between ChatGPT’s accuracy and the increasing difficulty of questions. This trend is anticipated and offers reassurance that the AI’s struggles are congruent with those encountered by human examinees. Notably, ChatGPT tends to correctly answer questions that a majority of test takers respond to accurately. Although ChatGPT generally surpasses trainee performance benchmarks, its accuracy aligns predictably with the variation in question difficulty.

### 4.3. Impact of Domain-Specific Pretraining

Domain-specific pretraining enables the model to improve its score by an average of 7%, from 78% to 85% overall. The domain-specific pretrained ChatGPT Plus model also performed better across all test sections, except in pediatrics and strabismus, where the performance was identical. The most notable improvements were observed in glaucoma and clinical optics, two sections where the non-pretrained ChatGPT struggled more than other sections. This indicates that increased difficulty allows ChatGPT Plus with pretraining to significantly outperform its counterpart. This phenomenon is depicted in Figure 2, which shows the widening accuracy gap between both models as question difficulty increases.

In other words, although both models achieved similar results for easy questions, the pretrained one surpassed its counterpart by 8% for moderate questions and by 27% for hard questions. These findings demonstrate the usefulness of domain specific pretraining and highlight how its addition filled gaps in the bot’s knowledge and rendered it capable of correctly answering increasingly challenging questions. However, some questions were repeatedly answered incorrectly by ChatGPT, even after pretraining. This indicates that these questions may either be flawed or unusually complex, suggesting a limitation in ChatGPT’s advanced problem-solving despite additional information. Therefore, simply providing more knowledge is not a guaranteed method to improve its performance.

Regardless, pretraining a ChatGPT model in specialized fields like ophthalmology offers a multitude of significant advantages, as the pretraining process involves exposing the model to a vast array of text data, enabling it to internalize a broad spectrum of language patterns and domain-specific knowledge [37,38]. In ophthalmology, this means that the pretrained model arrives equipped with a foundational understanding of ophthalmic terminology, concepts, and practices sourced from authoritative texts like medical textbooks and widely-accepted clinical guidelines [25]. This comprehensive knowledge transfer empowers the model to offer more effective interactions and responses within the specialized domain.

### 4.4. Repeatability and Consistency

Our study underscores the repeatability and consistency of ChatGPT-4 Plus’s responses, demonstrated by kappa coefficients ranging from 0.79 to 0.95 across individual sections. With substantial to almost perfect agreement observed in most sections, ChatGPT-4 Plus exhibits a high level of consistency in providing similar responses when presented with identical questions three separate times. Despite this, there have been reports of inconsistent behavior in ChatGPT-4 over time [29]. Thus, while our testing period revealed near-perfect repeatability and consistency, the model’s responses could vary with time, such as over several months, suggesting a dynamic aspect to its performance. This variability introduces important considerations for the long-term reliability of LLMs and their potential integration into clinical workflows, where consistent performance is crucial. Nevertheless, this evolving nature of ChatGPT’s responses might not be entirely negative. It could simply indicate the model’s ongoing knowledge updates and adaptations, reflecting its ability to incorporate new information and databases over time.

### 4.5. ChatGPT-4 Plus’ Performance Against Average Test-Takers

GPT-4 outperformed historical human scores on the BCSC datasets, showing statistically significant superior scores both individually and collectively. ChatGPT’s superior performance can be attributed to its ability to process vast amounts of information, surpassing human memorization in recall-focused sections, and its consistency and immunity to fatigue, unlike human examinees who may be impacted by stress or tiredness. Historical scores on the BCSC datasets were derived from the combined performance of international ophthalmology trainees, including residents and fellows, on question banks, serving as a stand-in for human benchmarking. This approach is notable for its reliance on average scores drawn from the experiences of thousands of trainees worldwide over several years. It’s important to understand, though, that these question banks are primarily used as educational tools. As a result, the trainees might not always give their full effort when answering these questions, treating them as practice items without real-world consequences. This lack of full effort can manifest in several ways: trainees may not fully engage with the questions, might leave them unanswered, or submit their responses prematurely just to view the correct answers. Such behaviors do not accurately reflect the trainees’ true abilities and could lead to lower average scores. Despite this, the impact is somewhat balanced by the fact that trainees tend to perform better on subsequent attempts after becoming familiar with the questions.

### 4.6. Strengths and Limitations

Our study is, to the best of our knowledge as of this date, one of the first to incorporate questions with clinical images, setting it apart from previous research that excluded questions containing clinical images and focused solely on those without images. Additionally, this is a pioneering exploration of the impact of domain-specific pretraining on ChatGPT’s performance. This study also boasts a significant sample size of 1300 questions, amounting to 5200 fed questions/prompts when considering three repeats to test repeatability and one additional set for the pretraining model, with 100 questions per section randomly selected.

One limitation of our study is the restricted token capacity of ChatGPT-4, which may limit the exploration of its full potential in processing image-based questions, especially in the pretrained version. The extensive token requirement for image interpretation could lead to an underestimation of ChatGPT’s capabilities in image recognition [32]. Efforts were made to obtain the enterprise version of ChatGPT from OpenAI, which offers a higher token limit, but these attempts were unsuccessful. Another constraint is the absence of temperature adjustments. However, prior research has indicated that this parameter does not significantly affect the model’s accuracy [25]. Additionally, our study did not assess the cognitive level of questions due to the labor-intensive nature of analyzing the extensive dataset we presented to ChatGPT. Previous studies have already established a correlation between cognitive level and performance, where models tend to perform worse at higher levels of reasoning and complex problem solving [20,25]. Therefore, we chose to focus on parameters not previously explored by other studies, rather than on cognitive levels that have already been examined. Another limitation to note is that our study solely focused on multiple-choice questions from a single source, which may limit the generalizability of our results.

These limitations illustrate the numerous factors, in terms of both the AI’s setting and the characteristics of the questions, that affect the chatbot’s performance. Apart from the topic, presence of images, question difficulty, and addition of domain-specific pretraining, many more elements, such as the aforementioned aspects, need to be considered to paint a more complete picture of the AI’s capabilities. Further fine-tuning and parameter adjustments may have yielded slightly higher accuracies [39]. To do so, subsequent research should first aim to secure a more advanced model with increased token capacity to fully evaluate ChatGPT’s potential. Additionally, ensuing research should incorporate more image-based questions along with different types of questions, such as short answer, long answer, multiple selection, matching, and computational questions. Lastly, with the continuous advancements in AI technologies, future directions could also explore other computational methods to customize ChatGPT and optimize its performance for ophthalmology-specific tasks.

## 5. Conclusions

Our research delineates key findings as follows:

1. The ChatGPT Plus model demonstrated a superior performance, surpassing the average test-taker by a margin of 4%. When enhanced with domain-specific pretraining, this model further outshone the non-pretrained ChatGPT Plus variant by 7% and exceeded average test-taker scores by 11%.
2. The benefits of domain-specific pretraining become increasingly significant as the difficulty of questions increases. However, this advantage does not extend to performance on image-based questions.
3. The accuracy of ChatGPT-4’s responses is primarily influenced by the difficulty of questions, followed by the presence of images, and the specific exam section. ChatGPT scores higher on questions categorized as easy, those without images, and within certain sections, such as general medicine and fundamentals.
4. The repeatability and consistency of ChatGPT-4 Plus’s responses are affirmed by kappa coefficients spanning from 0.79 to 0.95 across individual sections, indicating a range from substantial to nearly perfect concordance.

This study marks the first of its kind to incorporate images, domain-specific pretraining, and a comprehensive dataset of 1300 questions. Our findings, notably the improvement seen with domain-specific pretraining as well as the image recognition capacity, are encouraging. Despite these promising results, we caution against viewing these achievements as indicative of ChatGPT-4 reaching the comprehensive skill level of a human ophthalmologist. Performance in multiple-choice exams, similar to human trainees, does not reflect the full spectrum of clinical competence. Qualities such as communication, collaboration, leadership, and professionalism, crucial for physicians, remain unmeasured by exam scores [40]. Additionally, recognizing the limits of one’s knowledge, a critical physician skill, poses challenges for generative AI due to its probabilistic nature [41].

Looking ahead, further research should explore the safety and efficacy of AI in clinical decision-making, extending beyond multiple-choice queries to include open-ended questions that encompass complex patient data. This would more accurately reflect clinical realities, assessing AI’s ability to interpret diverse information, formulate treatment plans, evaluate prognosis, and recognize when specialist referral is necessary. Enhancing AI’s capability to process multimodal inputs will also be key. Collaborations with AI firms like OpenAI to access more advanced models with increased token capacities are crucial for unlocking the full potential of generative AI in medicine.

## Figures and Tables

**Table 1:** Baseline characteristics of the testing sets.

	Difficulty Level			Presence of Images	
	Easy	Moderate	Difficult	Without Images	With Images
BCSC Overall (*N* = 1300)	459	751	90	1148	152
General Medicine (*N* = 100)	49	43	8	99	1
Fundamentals (*N* = 100)	35	60	5	97	3
Clinical Optics (*N* = 100)	22	66	12	96	4
Ophthalmic Pathology (*N* = 100)	21	59	20	85	15
Neuro-Ophthalmology (*N* = 100)	40	55	5	83	17
Pediatrics and Strabismus (*N* = 100)	30	68	2	91	9
Oculoplastics (*N* = 100)	44	56	0	86	14
Cornea (*N* = 100)	58	41	1	70	30
Uveitis (*N* = 100)	30	59	11	90	10
Glaucoma (*N* = 100)	31	65	4	91	9
Lens and Cataract (*N* = 100)	36	58	6	86	14
Retina and Vitreous (*N* = 100)	26	64	10	82	18
Refractive Surgery (*N* = 100)	37	57	6	92	8

**Table 2:** Cohen’s kappa coefficient by section.

Section	Cohen’s Kappa (κ)	95% CI
General medicine	0.9	0.83–0.97
Fundamentals	0.82	0.74–0.90
Clinical optics	0.8	0.71–0.89
Ophthalmic pathology	0.82	0.74–0.90
Neuro-ophthalmology	0.79	0.69–0.88
Pediatrics and strabismus	0.84	0.75–0.92
Oculoplastics	0.83	0.75–0.92
Cornea	0.87	0.79–0.94
Uveitis	0.95	0.90–0.99
Glaucoma	0.81	0.72–0.90
Lens and cataracts	0.88	0.80–0.95
Retina and vitreous	0.84	0.76–0.92
Refractive surgery	0.86	0.78–0.93
Overall	0.85	0.82–0.87

## Data Availability

Data is contained within the article.

## Abbreviations

The following abbreviations are used in this manuscript:

AAO	American Academy of Ophthalmology
AI	Artificial intelligence
BCSC	Basic and Clinical Science Course
CT	Computed tomography
LLM	Large language model
LR	Likelihood ratio
MRI	Magnetic resonance imaging
OCT	Optical coherence tomography
USMLE	United States Medical Licensing Examination

## Appendix A

### Appendix A.1

Cohen’s kappa (κ) Formula

The Cohen’s kappa coefficient is calculated as:

κ = (p_o_ − p_e_)*/*(1 − p_e_)

where:

p_o_ = Observed agreement (proportion of actual agreement between raters);

p_e_ = Expected agreement (proportion of agreement due to chance).

1. Observed Agreement (p_o_):

p_o_ = (TN + TP)/(TN + FP + FN + TP)

2. Expected Agreement (p_e_):

p_e_ = P_empirical + P_theoretical

where:

P_empirical = [(TN + FP)/N] × [(TN + FN)/N]

P_theoretical = [(FN + TP)/N] × [(FP + TP)/N]

N = TN + FP + FN + TP (total observations)

Abbreviations:

TP = True Positive, FP = False Positive;

TN = True Negative, FN = False Negative.

### Appendix A.2

Logistic Regression Formula

The logistic regression model is expressed as:

$$\log\frac{p}{1-p}=\beta_{0}+\beta_{1}x_{1}+\beta_{2}x_{2}+\ldots +\beta_{m}x_{m}$$

where:

*p* = Probability of the outcome occurring;

*β*_0_ = Intercept term;

*β*_1_ to *β_m_* = Coefficients for predictor variables X_1_ to X_m_.

## References

[1] Aldoseri A., Al-Khalifa K.N., Hamouda A.M. (2023). Re-Thinking Data Strategy and Integration for Artificial Intelligence: Concepts, Opportunities, and Challenges. Appl. Sci..

[2] Basu K., Sinha R., Ong A., Basu T. (2020). Artificial Intelligence: How Is It Changing Medical Sciences and Its Future?. Indian J. Dermatol..

[3] Tolsgaard M.G., Pusic M.V., Sebok-Syer S.S., Gin B., Svendsen M.B., Syer M.D., Brydges R., Cuddy M.M., Boscardin C.K. (2023). The Fundamentals of Artificial Intelligence in Medical Education Research: AMEE Guide No. 156. Med. Teach..

[4] Ting D.S.W., Pasquale L.R., Peng L., Campbell J.P., Lee A.Y., Raman R., Tan G.S.W., Schmetterer L., Keane P.A., Wong T.Y. (2019). Artificial Intelligence and Deep Learning in Ophthalmology. Br. J. Ophthalmol..

[5] Li Z., Wang L., Wu X., Jiang J., Qiang W., Xie H., Zhou H., Wu S., Shao Y., Chen W. (2023). Artificial Intelligence in Ophthalmology: The Path to the Real-World Clinic. Cell Rep. Med..

[6] Singh S., Djalilian A., Ali M.J. (2023). ChatGPT and Ophthalmology: Exploring Its Potential with Discharge Summaries and Operative Notes. Semin. Ophthalmol..

[7] Waisberg E., Ong J., Masalkhi M., Kamran S.A., Zaman N., Sarker P., Lee A.G., Tavakkoli A. (2023). GPT-4 and Ophthalmology Operative Notes. Ann. Biomed. Eng..

[8] Ray P.P. (2023). ChatGPT: A Comprehensive Review on Background, Applications, Key Challenges, Bias, Ethics, Limitations and Future Scope. Internet Things Cyber-Phys. Syst..

[9] Achiam J., Adler S., Agarwal S., Ahmad L., Akkaya I., Aleman F.L., Almeida D., Altenschmidt J., Altman S., OpenAI (2024). GPT-4 Technical Report. arXiv.

[10] Singh S. (2025). ChatGPT Statistics 2025—DAU & MAU Data [Worldwide]. DemandSage.

[11] ChatGPT in Ophthalmology: The Dawn of a New Era?|Eye. https://www.nature.com/articles/s41433-023-02619-4.

[12] Dossantos J., An J., Javan R. (2023). Eyes on AI: ChatGPT’s Transformative Potential Impact on Ophthalmology. Cureus.

[13] Tan T.F., Thirunavukarasu A.J., Campbell J.P., Keane P.A., Pasquale L.R., Abramoff M.D., Kalpathy-Cramer J., Lum F., Kim J.E., Baxter S.L. (2023). Generative Artificial Intelligence Through ChatGPT and Other Large Language Models in Ophthalmology: Clinical Applications and Challenges. Ophthalmol. Sci..

[14] Cappellani F., Card K.R., Shields C.L., Pulido J.S., Haller J.A. (2024). Reliability and Accuracy of Artificial Intelligence ChatGPT in Providing Information on Ophthalmic Diseases and Management to Patients. Eye.

[15] Madadi Y., Delsoz M., Lao P.A., Fong J.W., Hollingsworth T.J., Kahook M.Y., Yousefi S. (2024). ChatGPT Assisting Diagnosis of Neuro-Ophthalmology Diseases Based on Case Reports. Journal of Neuro-Ophthalmology.

[16] Delsoz M., Madadi Y., Munir W.M., Tamm B., Mehravaran S., Soleimani M., Djalilian A., Yousefi S. (2024). Performance of ChatGPT in Diagnosis of Corneal Eye Diseases. Cornea.

[17] Shemer A., Cohen M., Altarescu A., Atar-Vardi M., Hecht I., Dubinsky-Pertzov B., Shoshany N., Zmujack S., Or L., Einan-Lifshitz A. (2024). Diagnostic Capabilities of ChatGPT in Ophthalmology. Graefes Arch Clin Exp Ophthalmol.

[18] Haddad F., Saade J.S. (2024). Performance of ChatGPT on Ophthalmology-Related Questions Across Various Examination Levels: Observational Study. JMIR Med. Educ..

[19] Sakai D., Maeda T., Ozaki A., Kanda G.N., Kurimoto Y., Takahashi M. (2023). Performance of ChatGPT in Board Examinations for Specialists in the Japanese Ophthalmology Society. Cureus.

[20] Antaki F., Touma S., Milad D., El-Khoury J., Duval R. (2023). Evaluating the Performance of ChatGPT in Ophthalmology: An Analysis of Its Successes and Shortcomings. Ophthalmol. Sci..

[21] Mihalache A., Popovic M.M., Muni R.H. (2023). Performance of an Artificial Intelligence Chatbot in Ophthalmic Knowledge Assessment. JAMA Ophthalmol..

[22] Balci A.S., Yazar Z., Ozturk B.T., Altan C. (2024). Performance of Chatgpt in Ophthalmology Exam; Human versus AI. Int. Ophthalmol..

[23] Balas M., Mandelcorn E.D., Yan P., Ing E.B., Crawford S.A., Arjmand P. (2025). ChatGPT and Retinal Disease: A Cross-Sectional Study on AI Comprehension of Clinical Guidelines. Can. J. Ophthalmol..

[24] Landis J.R., Koch G.G. (1977). The Measurement of Observer Agreement for Categorical Data. Biometrics.

[25] Antaki F., Milad D., Chia M.A., Giguère C.-É., Touma S., El-Khoury J., Keane P.A., Duval R. (2024). Capabilities of GPT-4 in Ophthalmology: An Analysis of Model Entropy and Progress towards Human-Level Medical Question Answering. Br. J. Ophthalmol..

[26] Cai L.Z., Shaheen A., Jin A., Fukui R., Yi J.S., Yannuzzi N., Alabiad C. (2023). Performance of Generative Large Language Models on Ophthalmology Board-Style Questions. Am. J. Ophthalmol..

[27] Mihalache A., Huang R.S., Popovic M.M., Muni R.H. (2023). Performance of an Upgraded Artificial Intelligence Chatbot for Ophthalmic Knowledge Assessment. JAMA Ophthalmol..

[28] Teebagy S., Colwell L., Wood E., Yaghy A., Faustina M. (2023). Improved Performance of ChatGPT-4 on the OKAP Examination: A Comparative Study with ChatGPT-3.5. J. Acad. Ophthalmol. (2017).

[29] Chen L., Zaharia M., Zou J. (2024). How Is ChatGPT’s Behavior Changing over Time?. Harv. Data Sci. Rev..

[30] Koga S., Du W. (2025). From Text to Image: Challenges in Integrating Vision into ChatGPT for Medical Image Interpretation. Neural Regen. Res..

[31] Roumeliotis K.I., Tselikas N.D. (2023). ChatGPT and Open-AI Models: A Preliminary Review. Future Internet.

[32] Kumar M. (2023). Understanding Tokens in ChatGPT. Medium.

[33] Zhou Y., Chia M.A., Wagner S.K., Ayhan M.S., Williamson D.J., Struyven R.R., Liu T., Xu M., Lozano M.G., Woodward-Court P. (2023). A Foundation Model for Generalizable Disease Detection from Retinal Images. Nature.

[34] Wu X.-K., Chen M., Li W., Wang R., Lu L., Liu J., Hwang K., Hao Y., Pan Y., Meng Q. (2025). LLM Fine-Tuning: Concepts, Opportunities, and Challenges. Big Data Cogn. Comput..

[35] Kourounis G., Elmahmudi A.A., Thomson B., Hunter J., Ugail H., Wilson C. (2023). Computer Image Analysis with Artificial Intelligence: A Practical Introduction to Convolutional Neural Networks for Medical Professionals. Postgrad. Med. J..

[36] Mosqueira-Rey E., Hernández-Pereira E., Alonso-Ríos D., Bobes-Bascarán J., Fernández-Leal Á. (2023). Human-in-the-Loop Machine Learning: A State of the Art. Artif. Intell. Rev..

[37] Gu Y., Tinn R., Cheng H., Lucas M., Usuyama N., Liu X., Naumann T., Gao J., Poon H. (2022). Domain-Specific Language Model Pretraining for Biomedical Natural Language Processing. ACM Trans. Comput. Healthc..

[38] Pattam A. (2023). Generative AI: Episode #9: The Rise of Domain-Specific Large Language Models. Medium.

[39] Chen J.S., Reddy A.J., Al-Sharif E., Shoji M.K., Kalaw F.G.P., Eslani M., Lang P.Z., Arya M., Koretz Z.A., Bolo K.A. (2024). Analysis of ChatGPT Responses to Ophthalmic Cases: Can ChatGPT Think like an Ophthalmologist?. Ophthalmol. Sci..

[40] Kassam A., Cowan M., Donnon T. (2016). An Objective Structured Clinical Exam to Measure Intrinsic CanMEDS Roles. Med. Educ. Online.

[41] Dave T., Athaluri S.A., Singh S. (2023). ChatGPT in Medicine: An Overview of Its Applications, Advantages, Limitations, Future Prospects, and Ethical Considerations. Front. Artif. Intell..

